# Structure-Activity Relationship of Pine Nut-Derived Peptides and Their Protective Effect on Nerve-Cell Mitochondria

**DOI:** 10.3390/foods11101428

**Published:** 2022-05-15

**Authors:** Hongyan Lu, Li Fang, Xiyan Wang, Dan Wu, Chunlei Liu, Xiaoting Liu, Ji Wang, Yawen Gao, Weihong Min

**Affiliations:** 1 College of Food Science and Engineering, Jilin Agricultural University, Changchun 130118, China; luhongyan0813@163.com (H.L.); fangli1014@126.com (L.F.); wangxiyan199294@163.com (X.W.); dandan84821677@163.com (D.W.); liuchunlei0709@jlau.edu.cn (C.L.); liuxiaoting@jlau.edu.cn (X.L.); wangji198644@163.com (J.W.); feng.1997@163.com (Y.G.); 2 National Engineering Laboratory of Wheat and Corn Deep Processing, Changchun 130118, China

**Keywords:** structure-activity relationship, antioxidative activity, active peptides, molecular docking, mitochondrial function

## Abstract

This study aimed to investigate the structure-activity relationship of the pine nut antioxidant peptide WYPGK and its derivative peptides, and to evaluate the protective effect of the latter on oxidative damage to mitochondrial structure and function in PC12 cells. Molecular docking revealed the derivative peptides WYFGK and WYSGK to have higher affinity to the active region of sirtuin 3 (SIRT3) (−6.08 kcal/mol and −5.87 kcal/mol, respectively), hence indicating that they are promising SIRT3 inducers and antioxidant factors. The derivative peptide WYSGK presented the highest ORAC value (5457.70 µmol TE/g), ABTS scavenging activity (70.05%), and Fe^2+^-chelating activity (81.70%), followed by WYPGK and WYFGK. Circular dichroism and nuclear magnetic resonance data suggested that the presence of 3-Ser in WYSGK increased its β-sheet content, and that the active hydrogen atoms produced chemical shifts. In H_2_O_2_-induced PC12 cells, WYSGK substantially reduced ROS and MDA levels, and increased ATP levels. Transmission electron microscopy and Seahorse Analyze assay proved the peptide WYSGK to significantly alleviate mitochondrial damage and respiratory dysfunction (*p* < 0.05), thereby implying that a study of structure-activity relationships of the peptides can possibly be an effective approach for the development of functional factors.

## 1. Introduction

Oxidative stress can cause various types of damage to cells, including DNA breakage, protein inactivation, and cell membrane damage. Excessive reactive oxygen species (ROSs) accumulation in the body can cause cytotoxicity, resulting in many diseases, such as tumors, Alzheimer’s disease, and diabetes [1,2]. Mitochondria are the main sites of redox reactions and energy metabolism in cells and are also the primary targets of ROS production and attack [3]. Damaged mitochondria cause increased oxidative stress and disturbance of energy metabolism, thereby damaging mitochondrial morphology and causing mitochondrial homeostatic imbalance [4]. Studies have shown that both mitochondrial morphology and homeostasis are essential for maintaining mitochondrial dynamics and normal cell function [5]. Mitochondria are abundantly distributed in nerve cells. Therefore, to maintain mitochondrial health as the starting point of a neuroprotective strategy, supplementation with antioxidants, and maintenance of a balance between the body’s oxidation and antioxidant systems, would be of great value.

Recently, antioxidant peptides have drawn extensive attention as functional foods and supplements, although their structure-activity relationships and antioxidant mechanisms have not yet been fully elucidated. Many researchers have inferred that the sequence and position of amino acids in the peptides may affect the peptide structure and properties, thereby affecting their antioxidant activity [6]. Yang et al. reported that the soybean protein peptide SHECN had a higher β-sheet content and lower α-helix content, contributing to its antioxidant activity [7]. Ma et al. designed decapeptides Gly-Ala-Gly-Leu-Pro-Gly-Lys-His-Glu-Arg and Gly-Ala-Gly-Leu-Pro-Gly-Lys-Arg-Glu-His to demonstrate the influence of different amino acid positions on the antioxidant activity of peptides [8]. In addition, a recent study has shown the chemical groups on amino acid residues to be related to the antioxidant activity of peptides. For example, the phenolic hydroxyl group in Tyr, the indole group in Trp, and the imidazole group in His of the peptide may increase its interaction with free radicals [9]. Ajibola et al. had previously reported that the hydrophobic and aromatic amino acids (Phe, Tyr, and Trp) contained in a peptide can positively affect its antioxidant activity [10]. Liu et al. proved the amino acid properties in the C-terminal and N-terminal regions of antioxidant peptides to be related to their antioxidant activity [11]. Nwachukwu et al. proposed that N-terminal amino acids, with greater hydrophobicity in an active peptide, can significantly enhance the antioxidant activity of the peptide [12]. Although these studies may provide evidence that amino acid composition of the peptide, and N-terminal or C-terminal location of an amino acid in sequence, affects the antioxidant activity of the peptide, the effect of an amino acid located within the peptide sequence, and that of interaction between amino acid residues, in terms of antioxidant capacity, have not yet been determined.

Sirtuin 3 (SIRT3) is a deacetylase in the mitochondria that regulates the antioxidant system. It has a conserved enzyme core (aa126-399), which contains the binding site of SIRT3 substrates, and is responsible for deacetylation [13]. Previously, the peptide Trp-Tyr-Pro-Gly-Lys (WYPGK) had been identified from an enzymatic hydrolysate of pine nut (*Pinus koraiensis* Sieb. et Zucc.) albumin. Our previous studies had shown WYPGK to have a high antioxidant capacity and to activate SIRT3 in order to enhance synaptic plasticity and improve learning and memory in mice [14]. Pro has a unique cyclic structure and fixed ψ angle, giving proline a special conformational rigidity, relative to other amino acids, affecting its secondary structure in peptide sequences. In this study, based on the pine nut antioxidant peptide WYPGK, Pro was separately replaced by 19 other common amino acids to explore the resultant structure-activity relationship. Molecular docking analysis was used to screen the active peptides with high affinity to SIRT3, and the potent derivative peptides were verified by in-vitro chemical experiments. Circular dichroism (CD) and nuclear magnetic resonance (NMR) spectroscopy were used to analyze the secondary structures and active hydrogen atoms in the derived peptides. Effects of the derived peptides on the mitochondrial structure and function of cell models of oxidative stress were evaluated. Our study provided new insights into the structure-activity relationship and antioxidant mechanisms of antioxidant peptides.

## 2. Materials and Methods

### 2.1. Materials and Reagents

The peptides WYPGK, WYSGK, and WYFGK were synthesized by Jiangsu Ji Tai Peptide Industry Science and Technology Co., Ltd. (Yancheng, China); their purities were determined to be 99.18, 98.29, and 98.92%. The 2,2-azino-bis (3-ethylbenzothiazoline-6-sulphonic acid) diammoniumsalt (ABTS) and 2,2′-azobis-(2-amidinopropane) dihydrochloride (AAPH) were obtained from Sigma Aldrich (St. Louis, MO, USA). The rat pheochromocytoma PC12 cells were purchased from Zhong Qiao Xin Zhou Biotechnology Co., Ltd. (Shanghai, China). Fetal bovine serum (FBS) and RPMI 1640 medium were obtained from Gibco-BRL (Gaithersburg, MD, USA). The bicinchoninic acid kit to measure protein concentration were obtained from Nanjing Jiancheng Bioengineering Institute (Nanjing, China). Assay kits to detect reactive oxygen species (ROSs), microscale malondialdehyde (MDA), and adenosine 5′-triphosphate (ATP) were obtained from Beyotime Institute of Biotechnology (Shanghai, China).

### 2.2. Molecular Docking

Molecular docking is an important method to explore intermolecular interactions. The crystal structure of SIRT3 (PDB ID 3GLS) was obtained from the Protein Data Bank (www.rcsb.org; accessed on 1 July 2021). The structures of the peptides were generated by Discovery Studio 2019. The molecular docking calculations between SIRT3 and the peptides were performed using AutoDock. A total of 150 independent runs were carried out with a maximum of 25,000,000 energy evaluations and a population size of 300. A grid box of dimensions (70 × 70 × 70), with a spacing of 0.375 Å, was created. Lamarckian genetic algorithm (LGA) was applied for the docking calculations. The binding affinity of the peptide to SIRT3 was evaluated by binding energy, and the results were expressed as binding energy (kcal/mol). The clusters were ranked according to the lowest representative energy from each cluster. The lowest energy conformation in the most populated cluster was chosen for further study. Discovery Studio software analyzes hydrogen bonds between residues at the SIRT3 binding site.

### 2.3. Antioxidant Activity Assay

#### 2.3.1. Oxygen Radical Absorbance Capacity (ORAC) Assay

The modified ORAC assay was conducted according to the method of Dávalos, Gómez-Cordovés, and Bartolomé [15]. First, 25 μL sodium phosphate buffer (75 mM pH 7.4), 25 μL Trolox standard solution (12.5, 25, 50 and 100 μM) and 25 μL peptides (100 μM) were added to the 96-well black plate, respectively. Then, 150 μL of fluorescein (63 nM) was added to each well, and the mixture was incubated at 37 °C for 30 min. After that, 25 μL of AAPH solution was added rapidly; the fluorescence was continuously measured with excitation wavelength 485 nm and emission wavelength 538 nm at 37 °C for every 2 min, until it reached 5% of the initial fluorescence intensity. The ORAC values were expressed as μmol Trolox equivalent/g (μmol TE/g) peptide.

#### 2.3.2. ABTS Assay

The modified ABTS assay was conducted according to the method of Re et al. [16]. Briefly, the ABTS stock solution was prepared using 7 mM ABTS and 2.45 mM potassium persulfate at a 1:1 (*v*:*v*) proportion for 12–16 h in the dark. The ABTS stock solution was diluted to 0.70 ± 0.02 absorbance at 734 nm wavelength by 5 mM phosphate-buffered saline (PBS, pH 7.4), which was used as the working solution. Then, 10 μL of peptides (100 µM) and 190 μL of ABTS working solution were added into the 96-well microplate. After 6 min, the absorbance was assayed at 734 nm wavelength. The ABTS radical scavenging activity was calculated as follows:

$$\mathrm{ABTS}\;\mathrm{radical}\;\mathrm{scavenging}\;\mathrm{activity}\;(\%)=\left(1-\frac{A_{1}-A_{2}}{A_{0}}\right)\times 100$$

where *A*_0_ is the control absorbance, *A*_1_ is the sample absorbance, and *A*_2_ is the blank absorbance.

#### 2.3.3. Fe^2+^-Chelating Activity Assay

Fe^2+^-chelating activity assay was conducted according to the method of Zhang et al. [17], with a slight modification. Briefly, 0.5 mL of peptides (100 µM), 1 mL of FeCl_2_ (20 µM) and 1 mL of ferrozine (0.5 mM) were added in the reaction. The mixture was uniformly mixed in a water bath at 25 °C for 20 min, and the absorbance was measured at a wavelength of 562 nm. The same concentration of EDTA was taken as a control. The Fe^2+^-chelating activity was calculated as follows:

$${\mathrm{Fe}}^{2+}\text{-}\mathrm{chelating}\;\mathrm{activity}\;\left(\%\right)=\left(1-\frac{A_{1}-A_{2}}{A_{0}}\right)\times 100$$

where *A*_0_ is the control absorbance, *A*_1_ is the sample absorbance, and *A*_2_ is the blank absorbance.

### 2.4. CD Measurements

The secondary structure determination of peptides was performed using a J-1500CD spectrometer (Spectroscopic Co., Ltd., Tokyo, Japan). The peptide solution at a concentration of 0.5 mg/mL was added to a quartz dish with a 0.1 mm optical path length. The spectral range was set to 190–260 nm, with a scanning speed of 100 nm/min and a response time of 2 s. The scanning was performed 3 times. The secondary structure content was calculated using the Reed′s Reference method.

### 2.5. NMR Measurements

NMR spectroscopy was conducted according to the method of Delaglio et al. [18]. A Bruker Avance III 500 MHz NMR spectrometer (Bruker BioSpin GmbH, Rheinstetten, Germany) was used to acquire 1H NMR spectra, COSY spectra, and NOESY spectra using a 5 mm broad band fluorine observation probe (BBFO) with z-gradients. The peptides were dissolved in 600 μL of D_2_O to analysis, and its 1H NMR spectra were obtained. Then, 10 mg of peptides were dissolved in 600 μL of DMSO solution and transferred to a 5 mm NMR tube, and the COSY and NOESY spectra were obtained. The main operating parameters were the sampling delay time of 6 s and the pulse width of 13 μs, which were recorded 32 times in total.

### 2.6. Cell Culture

PC12 cells were maintained in RPMI-1640 medium (Gibco-BRL, Gaithersburg, MD, USA), which was supplemented with 10% FBS at 37 °C and 5% CO_2_. Cells were seeded at a density of 2 × 10^5^ cells/mL in 24- or 6-well plates, cultured with peptides for 24 h, and then exposed to concentration (0.4 mM) of H_2_O_2_ for 3 h. Cells in the control group were untreated, and cells in the model group were treated with H_2_O_2_.

### 2.7. Determination of ROS, MDA and ATP Levels

Fluorescent probe 2′,7′-dichlorodihydrofluorescein diacetate (DCFH-DA) was used to detect intracellular ROS. After incubation with peptides for 24 h and then with H_2_O_2_ for 3 h, 10 μM DCFH-DA was added to each well, and the cells were incubated at 37 °C for 30 min and washed twice with PBS to eliminate excess DCFH-DA. A fluorescence microscope (Carl Zeiss Jena GmbH, Jena, Germany) was used for photographs. Culture supernatant was collected and fluorescence was measured at excitation and emission wavelengths of 485 and 535 nm, respectively. Intracellular reactive oxygen levels were compared, based on relative fluorescence intensity.

For measurements of MDA and ATP levels, PC12 cells were cultured in 6-well plates, exposed to the peptides and H_2_O_2_, washed twice with ice-cold PBS, collected, lysed for 30 min in cell lysis buffer (Beyotime Biotechnology, Suzhou, China) at low temperature, and centrifuged. Protein concentration in supernatants was measured using the BCA kit, and MDA and ATP levels were determined according to the appropriate kit instructions.

### 2.8. Transmission Electron Microscopy (TEM) Measurements

After peptide treatment, the cells were trypsinized and fixed in 0.1 M PBS (pH 7.4) at 4 °C with 2% glutaraldehyde for 2 h. After washing three times with 0.1 M PBS, the cells were exposed to 1% tetraoxide for 2 h. Then, the tissue was dehydrated using an ethanol gradient. Subsequently, the samples were embedded in Epon Resin 812, cut to 60–80 nm thinness, and stained with uranium and lead. The TEM (H-7650, Hitachi, Tokyo, Japan) was used to observe the mitochondrial ultrastructure images.

### 2.9. Mitochondrial Respiration Measurements

The cellular oxygen consumption rate (OCR) and extracellular acidification rate (ECAR) were assessed using a Seahorse XF8 extracellular flux analyzer (Seahorse Bioscience, North Billerica, MA, USA). Briefly, PC12 cells were seeded at a density of 3 × 10^5^ cells into Seahorse XF cell plates for 24 h. After the peptides and H_2_O_2_ were treated, the cells were incubated with the SIRT3 inhibitor 3-TYP (50 µM) for 2 h. During the detection of OCR, the drugs oligomycin (ATP synthase inhibitor, 1 μM), FCCP (uncoupler, 0.5 μM), rotenone (complex I inhibitor, 1 μM), and antimycin A (complex III inhibitor, 1 μM) were added in sequence. During the detection of ECAR, the drugs glucose (10 mM), oligomycin (1 μM), or 2-DG (100 mM) were added in sequence. Seahorse software was used to plot the results.

### 2.10. Statistical Analysis

Results were quantified and expressed as means ± standard deviation (SD). All experiments were performed in triplicate. One-way ANOVA was performed using GraphPad Prism 6 (GraphPad Software Inc., San Diego, CA, USA), and the Tukey test was applied to multiple comparisons. Differences were considered significant at *p* < 0.05.

## 3. Results and Discussion

### 3.1. Construction and Screening of Pine Nut-Derived Peptides

**Figure 1:**
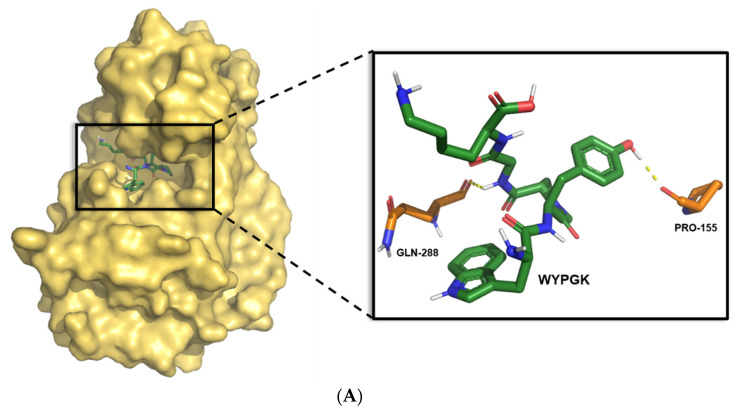

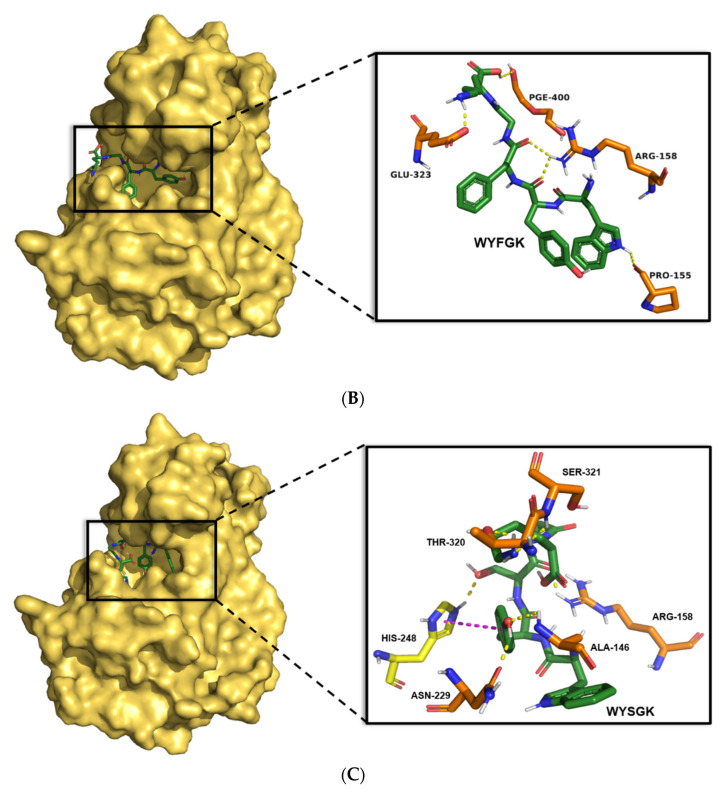

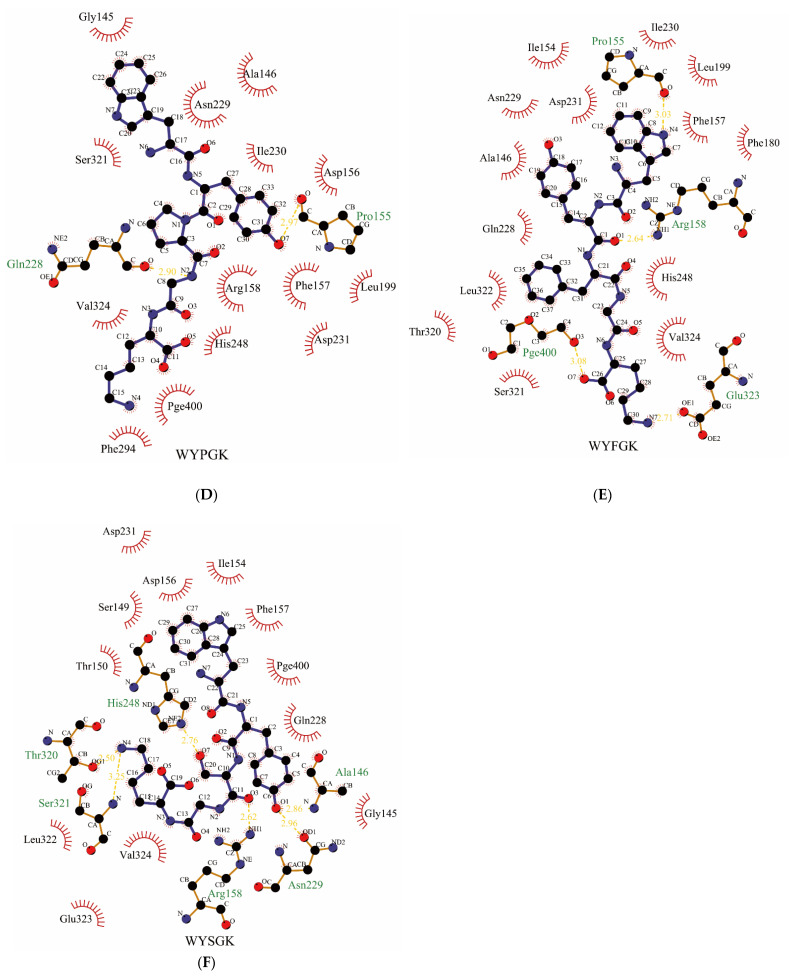
The molecular docking mode of (**A**) WYPGK, (**B**) WYFGK and (**C**) WYSGK with SIRT3. The yellow dashed lines stand for hydrogen bonds. The interaction between peptides and the residues of the binding sites in SIRT3 are shown using a 2 D diagram by LigPlus software. (**D**) WYPGK, (**E**) WYFGK and (**F**) WYSGK.

Molecular docking results of the antioxidant peptide WYPGK and its derivatives with the SIRT3 active region are shown in Table 1. Among the 20 peptides, WYFGK exhibited the lowest binding energy (−6.08 kcal/mol) followed by WYSGK (−5.87 kcal/mol), which was lower than that of WYPGK (−4.66 kcal/mol). It is widely accepted that the lower the binding energy of the protein-substrate docking, the stabler the complex. Therefore, the derivative peptides WYSGK and WYFGK with the lowest binding energy were selected for further study. The peptides WYPGK, WYFGK, and WYSGK might effectively interact with the SIRT3 active region and enhance deacetylation activity. Next, the influence of their amino acids on the structure-activity relationship of the active peptides was explored. The SIRT3-WYPGK complex structure obtained by molecular docking is shown in Figure 1A. Several strong hydrophobic interactions were found between WYPGK and the side chains of Gln228 and Pro155. The potential binding mode of WYFGK with SIRT3 is shown in Figure 1B. Pge400, Glu323, Pro155, and Arg158 seemed to be essential for maintaining the binding of WYFGK to SIRT3. His248, Asn229, Ala146, Thr320, Ser321, and Arg158 appeared to be important for the binding of WYSGK to SIRT3, as shown in Figure 1C. The imidazole ring of the His248 side chain was parallel to the phenolic hydroxyl plane of WYSGK tyrosine, implying that a strong π–π interaction existed between WYSGK and His248. A previous study had reported His248 to be critical for SIRT3 deacetylation activity [19]. Another study had reported the π-π interaction to be an important non-covalent interaction [20]. Thus, development of a π-π interaction might enhance the WYSGK and SIRT3 complex stability. As shown in Figure 1D, two hydrogen bonds exist in the SIRT3-WYPGK complex, namely O7-O (Pro155) and N2-O (Gln228). Four hydrogen bonds exist in the SIRT3-WYFGK complex, namely N4-O (Pro155), NH1-O (Arg158), O-O3(Pge400), and N-O(Glu323), as shown in Figure 1E. The 2D diagram (Figure 1F) showed SIRT3 and WYSGK to form five hydrogen bonds, namely O1-N (Ala146), O1-OD1 (Asn229), O3-NH1 (Arg158), O7-NE2 (His248), and N4-N (Ser321). Notably, arginine of the derived peptide WYSGK was anchored to the active site of SIRT3 via a special sandwich structure (Arg-158)/(WYSGK)/(His-248). In summary, compared to the SIRT3-WYSGK complex, the SIRT3-WYPGK complex and the SIRT3-WYFGK complex had weak binding stabilities. The SIRT3-WYSGK complex was speculated to show stable binding, with enhanced deacetylation activity, due to the formation of several hydrogen bonds and π-π interactions, along with sandwich structures in the active region.

### 3.2. Antioxidant Activity of the Pine Nut Peptide WYPGK and Its Derived Peptides WYFGK and WYSGK

**Figure 2:**
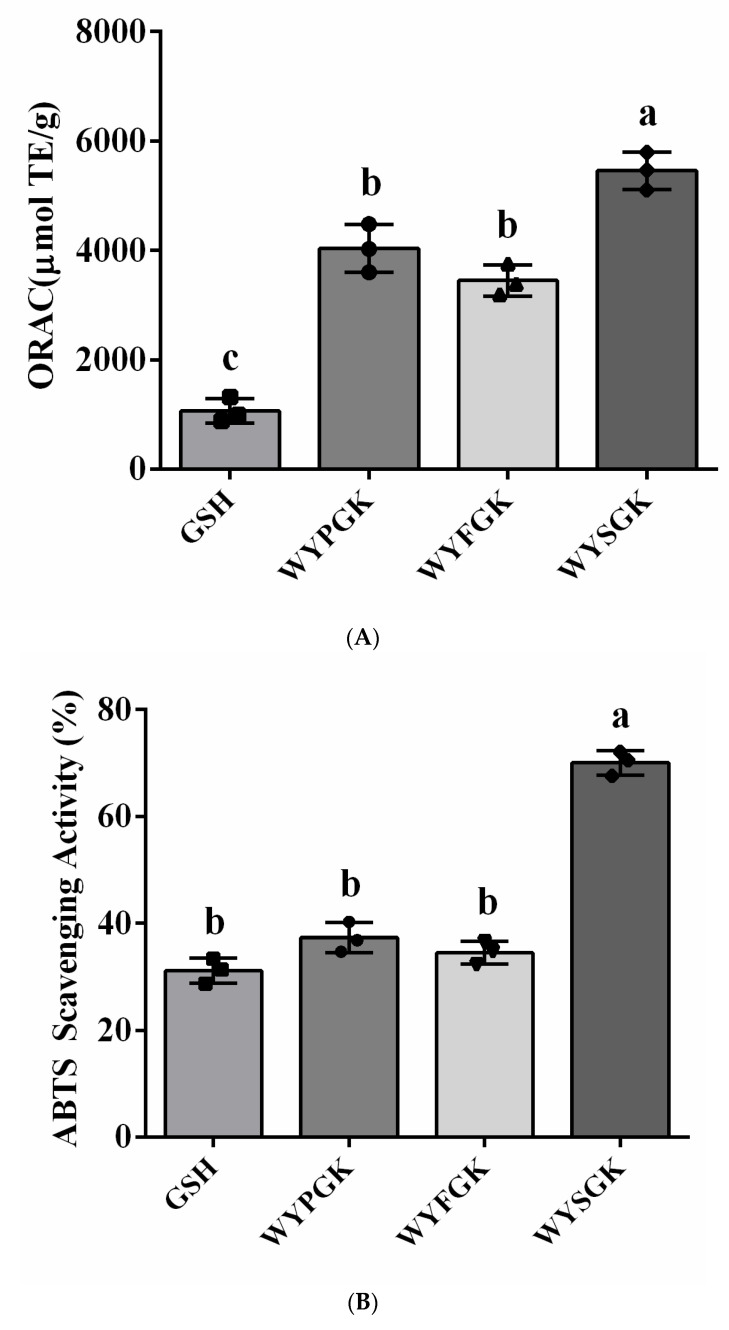

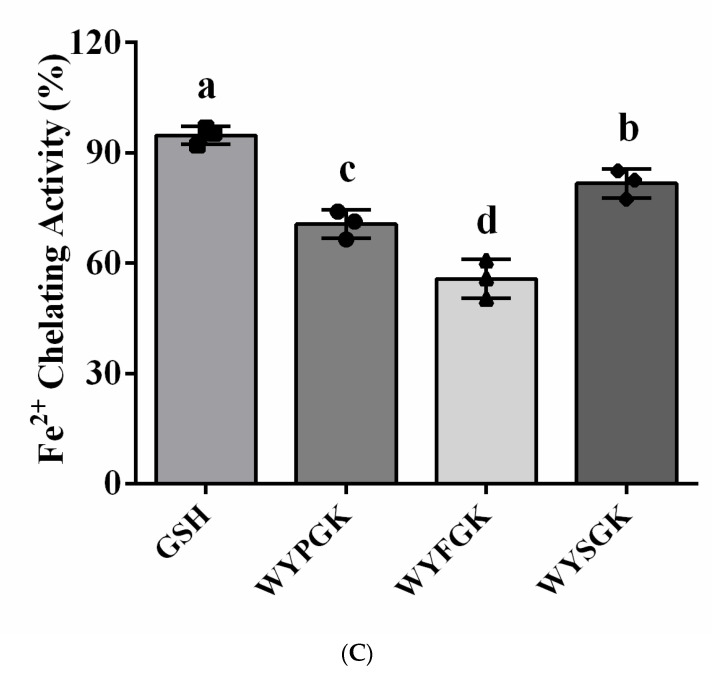
Antioxidant activity assay of WYPGK, WYFGK, and WYSGK. (**A**) ORAC assay; (**B**) ABTS radical scavenging activity; (**C**) Fe^2+^ chelating activity. Data are presented as means ± SD from three replicates. Different letters denote statistically significant differences (*p* < 0.05).

To confirm the antioxidant activity of the peptides, WYPGK, WYFGK, and WYSGK were chemically synthesized, and their activities determined using an in-vitro chemical assay. In the ORAC assay and ABTS free radical scavenging activity assay (Figure 2A,B), activity of the peptide-treated group was higher than that of the GSH group. Results demonstrated that the peptides exhibited high antioxidant capacity. A study had earlier reported that N-terminal bulky residues, such as Trp and Tyr, have strong radical scavenging activities [21]. Furthermore, basic amino acids (K, R, and H) were reported to act as hydrogen donors and have an effectively free radical scavenging activity [22]. Hence, an N-terminal Trp residue, a Tyr residue at the N-terminal second position, and a C-terminal Lys residue in five peptides could contribute to their antioxidant activity. The ORAC value of the WYSGK peptide was 5457.70 µmol TE/g, which was more than 1.35-fold higher than that of WYPGK (4040.22 µmol TE/g), and the ORAC value of the WYFGK peptide was 3452.18 µmol TE/g (Figure 2A). Significantly, in the ABTS free radical scavenging experiment, the free radical scavenging ability of WYSGK was significantly increased, whereas that of WYFGK was not significantly different (*p* < 0.05 compared to the WYPGK group; Figure 2B). As shown in Figure 2C, the Fe^2+^-chelating activities of WYPGK, WYFGK, and WYSGK were 70.67, 55.73, and 81.70%, respectively, which indicated that amino acids within the peptides significantly impact the antioxidant capacity. Habinshuti et al. had pointed out that tightness of the peptide structure affects its antioxidant property [23]. The aromatic side-chain residues of Phe in WYFGK are located inside the peptide sequence, and might affect the tightness of the peptide and reduce its antioxidant activity. The Glu carboxyl group next to Tyr in the octapeptide AEEEYPDL from Spanish dry-cured ham is believed to play a role in the peptide’s antioxidant activity, since it could induce the donation of a hydrogen atom from the phenolic hydroxyl in the tyrosine residue [24]. The amino acids inside the peptide were found to affect the peptide antioxidant activity, and serine gave the peptide more opportunities to combine with free radicals in the spatial structure, imparting a stronger antioxidant capacity. Thus, serine might influence the surrounding amino acids to combine with free radicals or metal ions, resulting in the greater antioxidant activity of WYSGK. According to the stability of the docking results of the derived peptides WYFGK and WYSGK with SIRT3, and the antioxidant capacity of the WYFGK and WYSGK, the results showed that the derivative peptide WYSGK had better antioxidant activity. Compared with the original peptide WYPGK, and in order to explore the effect of internal amino acids on the structure-activity relationship of peptides, we analyzed the secondary structure and active hydrogen atoms of WYPGK and the derivative peptide WYSGK.

### 3.3. Analysis of WYPGK and WYSGK by CD Spectroscopy

**Figure 3:**
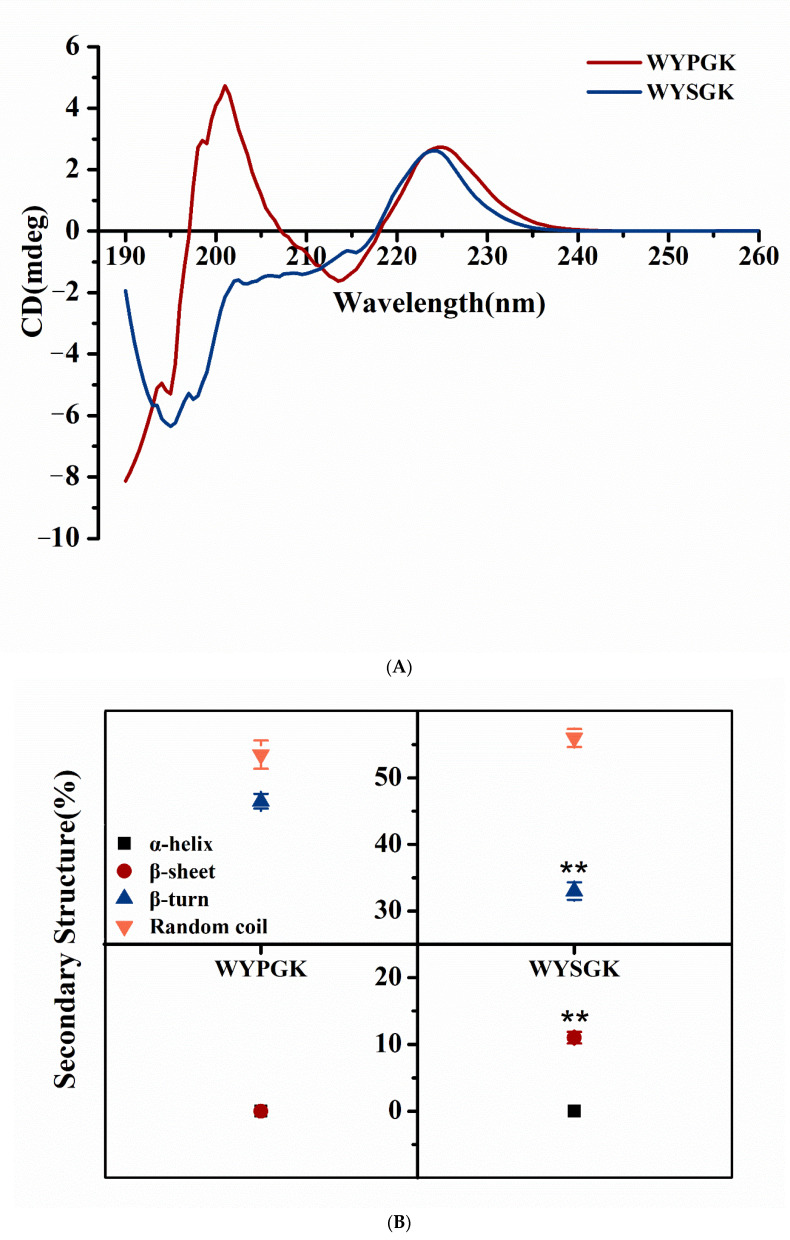

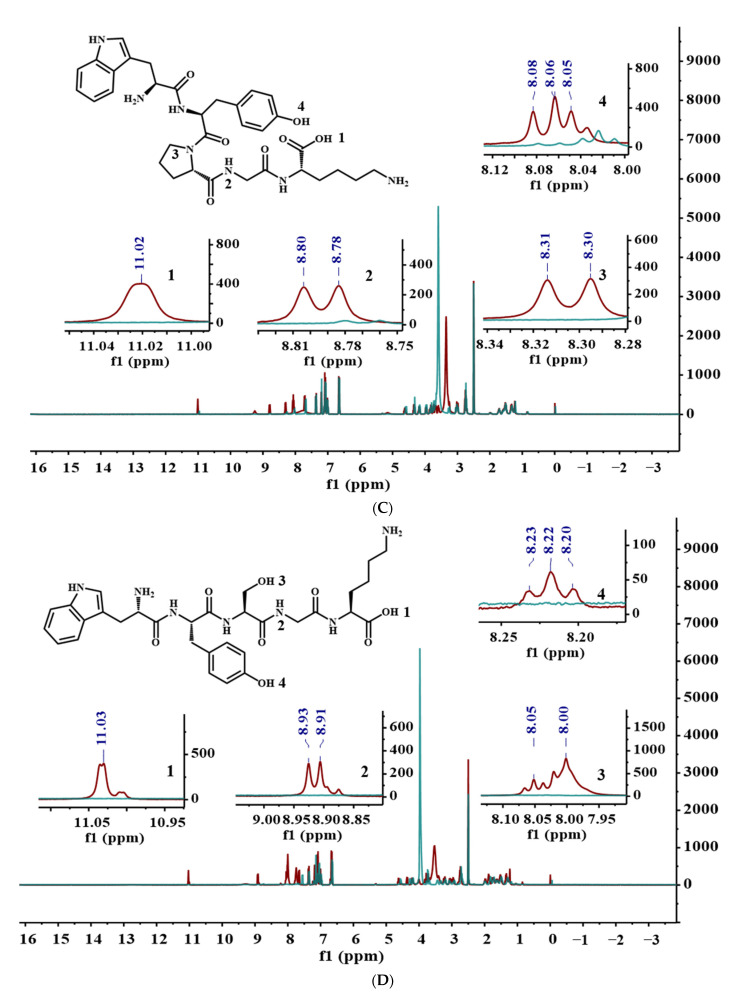

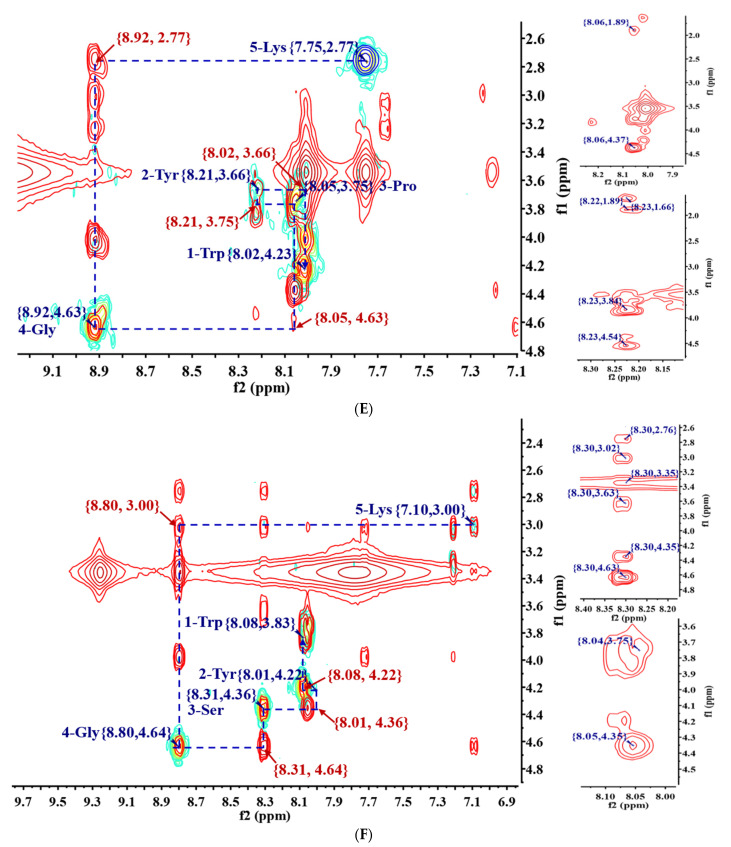
CD and NMR spectroscopy analysis of WYPGK and WYSGK. (**A**) CD spectra; (**B**) secondary structure analysis; the 1H NMR spectroscopy of (**C**) WYPGK and (**D**) WYSGK; COSY-NOESY NMR fingerprinting of (**E**) WYPGK and (**F**) WYSGK. ** indicates statistically significant differences (*p* < 0.01).

The CD spectra of WYPGK and WYSGK are shown in Figure 3A. WYPGK produced two positive bands around 225 nm and 200 nm each, whereas WYSGK produced a positive band near 225 nm, almost at the same position as WYPGK, and a negative band near 195 nm. Fric et al. proposed that the positive band at 225 nm is due to the presence of tyrosine at position 2 of the peptide sequence, which might explain why both the peptides WYPGK and WYSGK had a positive band at 225 nm [25]. Replacement of proline with serine in WYPGK changed the CD spectrum from a positive to a negative band around 200 nm, indicating that the substitution of amino acids at position N-3 affected the secondary structure of the peptide. To further clarify the influence on secondary structure, we analyzed the content of α-helices, β-sheets, β-turns, and random coil structures in WYPGK and WYSGK. Compared to that in WYPGK, the content of random coils in WYSGK increased from 53.5% to 56%, that of β-turn angle decreased from 46.5% to 33%, and a new β-sheet structure appeared with a content of 11% (Figure 3B). The results indicated that amino acid composition of the active peptide affected the peptide’s secondary structure. Liang et al. had shown that changes in the secondary structure of active peptides directly affect the antioxidant activity of peptides [26]. After ultrasound and enzymatic treatment of arrowhead protein, the content of β-sheets and random coils increased, and the antioxidant activity was significantly enhanced [27]. This was consistent with our previous research results; the increase in β-sheets and random coils in WYSGK could be the reasons underlying the enhanced antioxidant activity. The P residue located in the middle of the peptide sequence might cause the peptide chain to fold backward, promoting the formation of a β-sheet [28]. Therefore, we supposed that serine at the N-terminal third position might reduce the steric hindrance, due to its short side-chain group, expose the active site better, and exert antioxidant activity easily.

### 3.4. Analysis of WYPGK and WYSGK by NMR Spectroscopy

NMR spectroscopy is a general method for studying peptide-protein interactions in terms of binding affinity and binding kinetics; it shows the intramolecular and intermolecular interactions of peptide protons. ^1^H NMR spectrum analysis of the active hydrogen atoms of peptides WYPGK and WYSGK in the chemical environment were investigated here. Figure 3C shows the active hydrogen atoms of WYPGK and their corresponding chemical changes, as follows: −COOH (10.99–11.09 ppm), −CH_2_− (8.85–8.95 ppm), −N−CH_2_− (7.95–8.10 ppm), and −OH (8.15–8.25 ppm). The active hydrogen atoms in WYSGK and their corresponding chemical changes are shown in Figure 3D, as follows: −COOH (11.00–11.05 ppm), −CH_2_− (8.75–8.84 ppm), −OH (8.28–8.35 ppm), and −OH (8.00–8.14 ppm). We observed that in WYSGK, substitution of serine changes the active site of the active hydrogen atom (−N−CH_2_− is converted to −OH); the active hydrogen atom of the hydroxyl group on the serine side chain can be used as a hydrogen donor, which can possibly increase the antioxidant property of WYSGK. The unique cyclic structure of the proline side chain gives it a conformational rigidity, making it less free than other amino acids [29]. In-vitro ORAC, ABTS radical scavenging ability, and Fe^2+^-chelating activity experiments confirmed WYSGK to have stronger antioxidant activity than WYPGK. In addition, serine (S) substitution caused chemical shifts of the active hydrogen atoms on tyrosine (Y), glycine (G), and lysine (K), probably due to the different electronegativities of the bonding atoms. Therefore, WYSGK can scavenge free radicals, thereby acting as antioxidants.

Two-dimensional NMR COSY and NOESY patterns were used to analyze the close relationship across hydrogens in the spatial structure of WYPGK and WYSGK, and to further explore the relationship between peptide activity and configuration. The overlapping part of COSY spectrum and NOESY spectrum corresponds to the amino acid position, and the N_α_H−C_α_H chemical shift determines the position and arrangement of the amino acids. As shown in Figure 3E, the chemical shift positions of WYPGK 1-Trp, 2-Tyr, 3-Pro, 4-Gly, and 5-Lys have NOE connectivity of 8.02, 8.21, 8.05, 8.92, and 7.85 ppm, respectively, whereas in COSY, the peak values of connected C_α_H were 4.23, 3.66, 3.75, 4.63, and 2.77 ppm, respectively. Chemical shift positions of WYSGK (Figure 3F) indicated that the NOE connectivity of 1-Trp, 2-Tyr, 3-Ser, 4-Gly, and 5-Lys are 8.08, 8.01, 8.31, 8.80, and 7.10 ppm, whereas COSY was connected to C_α_H, the peaks being 3.83, 4.22, 4.36, 4.64, and 3.00 ppm, respectively. According to the NOESY spectrum, there is a methylene and methine proton assignment peak (−C_α_H) on 3-Pro and 3-Ser of the two peptides. In addition, there were five proton assignment peaks on the 3-Pro side-chain ring in WYPGK structure, which were 1.89, 4.37, 1.66, 3.84, and 4.54 ppm, respectively. In the structure of WYSGK, the assigned peaks of −C_β_H−OH on the side chain of 3-Ser were 4.63 and 4.35 ppm. The NOESY spectrum reflects the close relationship between the protons. The closer the protons, the greater would be their chance of contact with the outside world, and hence the greater would be the plasticity and activity of the peptide [30]. WYSGK had one less proton assignment peak than WYPGK, due to which it possessed more unrecognized protons that could bind to the active site through hydrogen bonds or other coordination bonds, and had a lower binding potential [31]. In addition, WYSGK had one more active hydrogen on the peptide bond -NH- and the terminal hydroxyl group. In summary, the presence of 3-Ser in WYSGK could be considered to increase the number of β-sheet active hydrogen atoms; since the peptide had a more extended and expanded spatial conformation, its chance of binding to the receptor was more, and so was its antioxidant activity.

### 3.5. Effects of WYPGK and WYSGK on ROS, MDA, and ATP Levels in H_2_O_2_-Treated PC12 Cells

**Figure 4:**
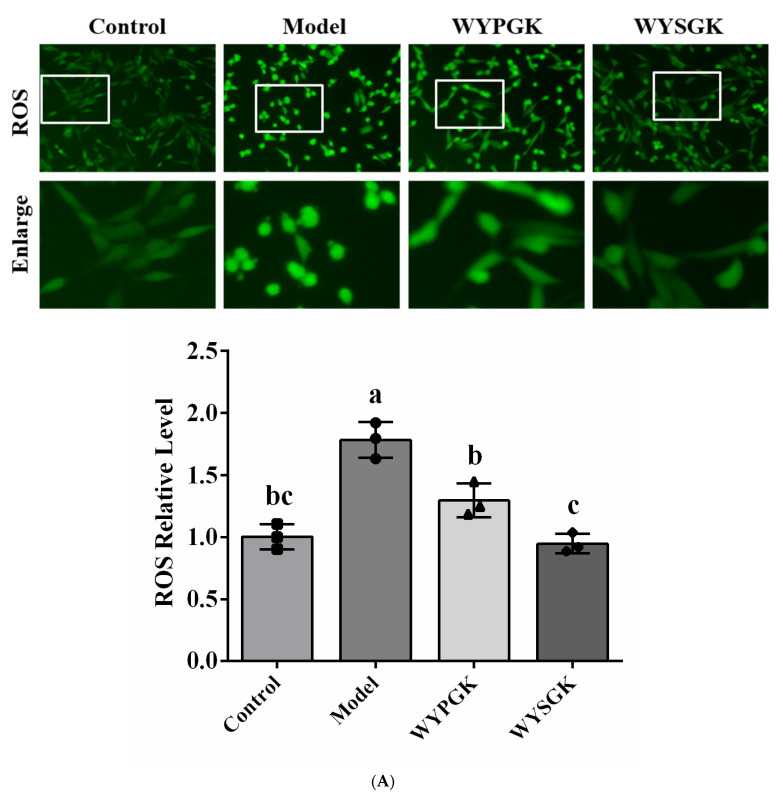

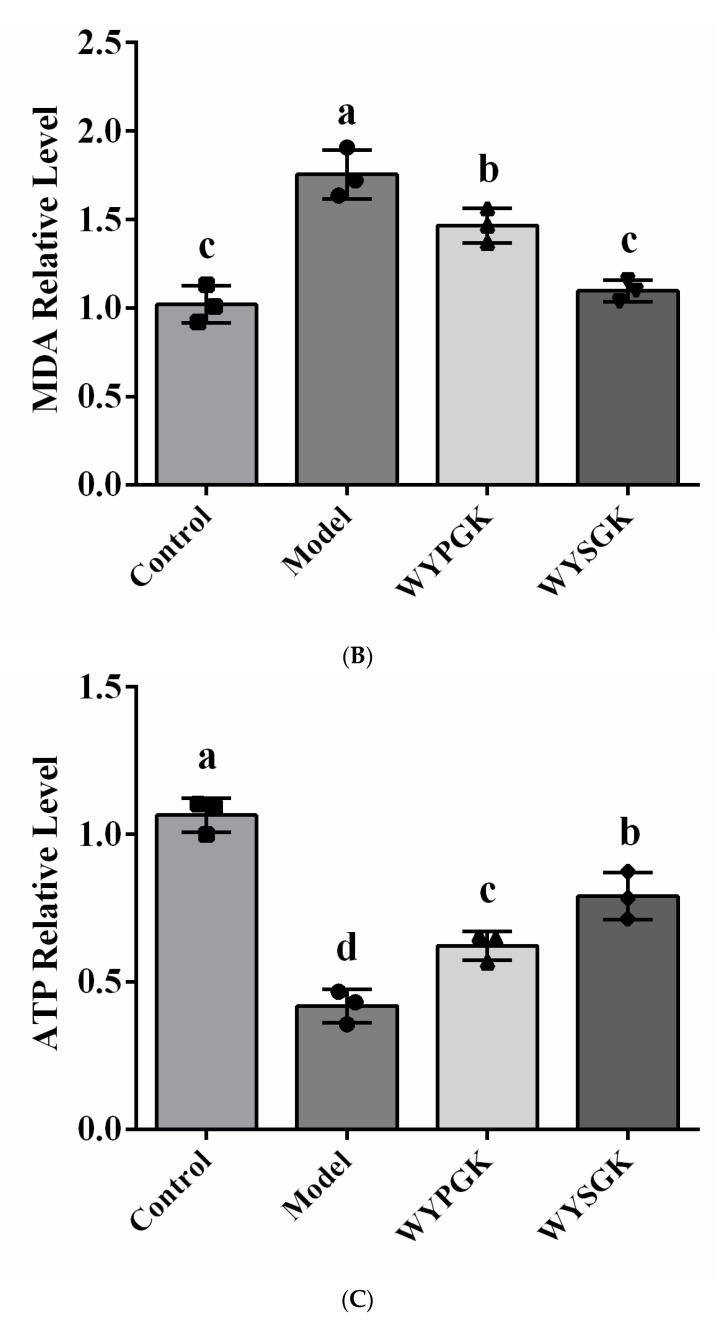

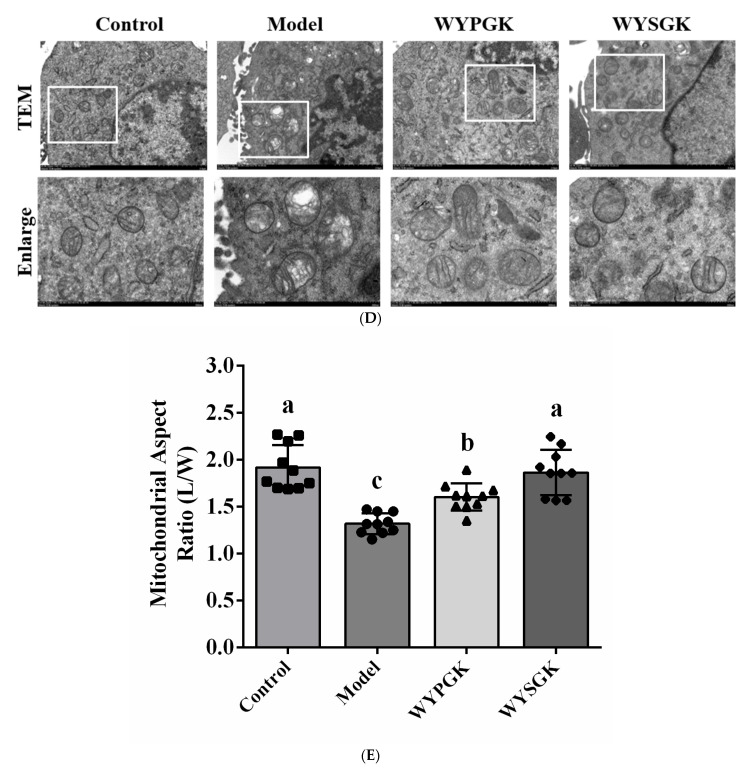
Effects of WYPGK and WYSGK on (**A**) ROS level; (**B**) MDA level; (**C**) ATP level; (**D**) mitochondrial structure and morphology; (**E**) mitochondrial aspect ratio. Representative quantitative results of mitochondrial length with 10 mitochondria per experiment. Data are presented as means ± SD from three replicates. Different letters denote statistically significant differences (*p* < 0.05).

The intracellular oxidized cell model is a common method used to assess the antioxidative capacity of compounds. H_2_O_2_ treatment significantly increased intracellular ROS levels (188.22%, Figure 4A) and MDA levels (187.27%, Figure 4B) compared to that in the control group (*p* < 0.05). However, ROS levels were significantly reduced in PC12 cells treated with the derivative peptides (*p* < 0.05). Pretreatment with WYSGK significantly decreased MDA levels in H_2_O_2_-induced PC12 cells (*p* < 0.05). Meanwhile, the ATP levels of WYPGK and WYSGK (0.64 ± 0.02 and 0.75 ± 0.09, respectively) were significantly increased over those of the model group (0.43 ± 0.03) (*p* < 0.05, Figure 4C). Compared to the presence of the phenyl ring of the phenylalanine side chain and the special cyclic structure of proline, the presence of serine in WYSGK could reduce steric hindrance of the polypeptide, simplifying the hydrogen bond network, and exposing more active sites, thereby showing a stronger antioxidant activity. Studies have pointed out that hydrogen bonds are an important force in the structure of proteins and peptides, maintaining the secondary structure of peptides and trapping free radicals, thereby influencing the antioxidant capacity of peptides [32]. This was consistent with the results of the secondary structure analysis of WYSGK.

### 3.6. Effects of WYPGK and WYSGK on Mitochondrial Damage in H_2_O_2_-Treated PC12 Cells

The protein encoded by SIRT3 exists only in the mitochondria, eliminating ROS and preventing neuronal damage. To evaluate the therapeutic effect of WYPGK, and its derivative peptide WYSGK, on abnormal mitochondria after oxidative damage, the ultrastructural changes in mitochondria were studied by transmission electron microscopy. As shown in Figure 4D, the mitochondria showed evident fragmentation, increased size, appearance of vacuoles, disappearance of the double-layer membrane structure, and breakage of the internal ridges in the model cell group. Compared to that in the model group cells, WYPGK treatment reduced the generation of vacuoles, and the internal crest was clearer. The WYSGK-treated group had the same therapeutic effect. Analysis of the aspect ratio of mitochondria (Figure 4E) suggested that the mitochondria of PC12 cells, damaged by H_2_O_2_, had morphological changes, as confirmed by decrease in the aspect ratio level. The mitochondrial aspect ratio was significantly restored after treatment with WYPGK and WYSGK, and that of the WYSGK treatment group was significantly higher than that of the WYPGK treatment group (*p* < 0.05). Lai et al. used the mitochondrial aspect ratio to analyze mitochondrial morphology and evaluate mitochondrial integrity quantitatively [33]. Sisalli et al. proved that the mitochondrial aspect ratio in hypoxic and glucose-deficient mouse neurons was significantly reduced, leading to mitochondrial dysfunction [34]. The results indicated that WYSGK treatment could maintain the function and integrity of mitochondria and effectively remodel the ultrastructure of mitochondria. Zhao et al. had demonstrated that the walnut peptide YVLLPSPK could significantly reduce neuronal mitochondrial damage and improve the damage in mitochondrial inner cristae [35]. Specific activation of the SIRT3 pathway could effectively improve mitochondrial dysfunction and promote mitochondrial development [36]. Consistent with our previous studies, WYSGK could intervene in the SIRT3 protein active pocket. In fact, WYSGK was speculated to possibly regulate mitochondrial function by mediating SIRT3 activation. In addition, the structure of mitochondrial cristae was shown to regulate mitochondrial respiratory function, indicating that the improved mitochondrial ultrastructure, induced by WYSGK, could be related to the bioenergy state. Compared with WYPGK, the derived peptide WYSGK showed better mitochondrial protection. Therefore, the derived peptide WYSGK was selected to further analyze the regulatory role of WYSGK on mitochondrial respiration and explore whether SIRT3 protein could be involved in mitochondrial respiration.

### 3.7. Effects of WYSGK on Mitochondrial Respiration

**Figure 5:**
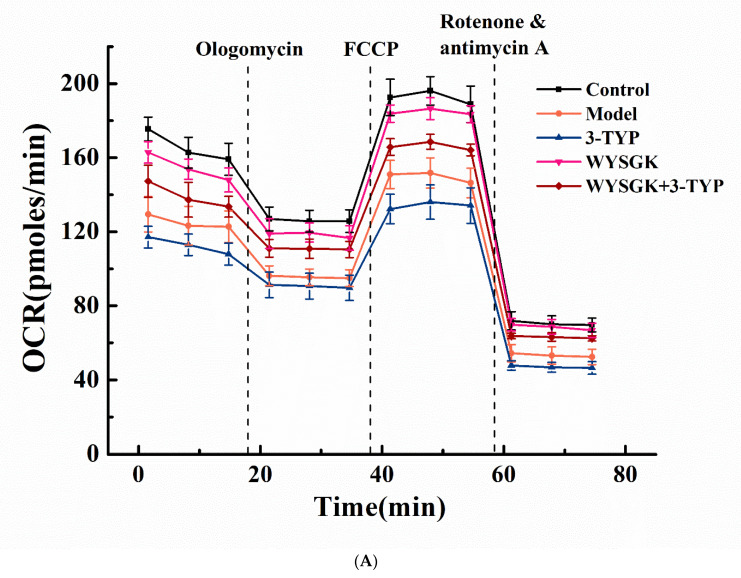

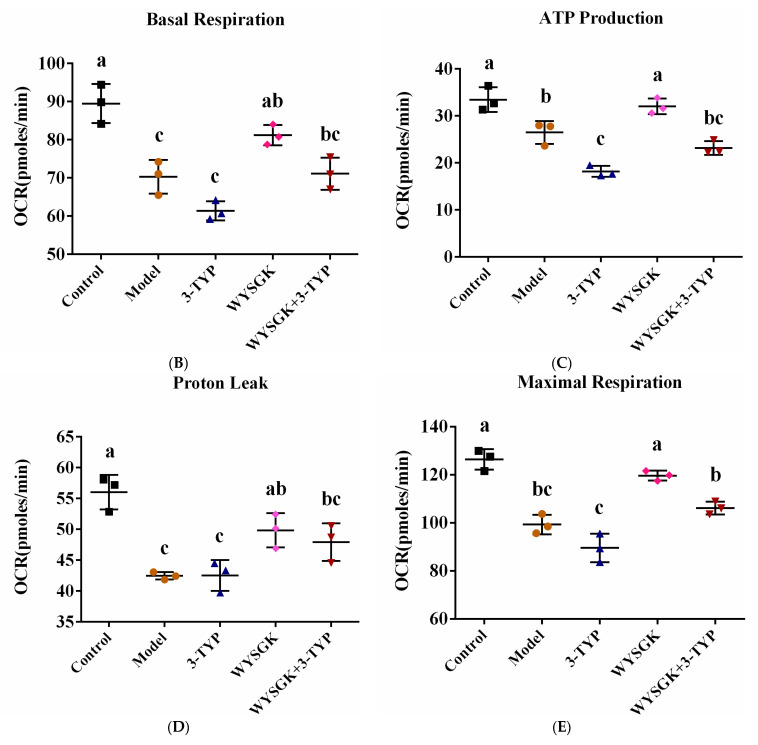

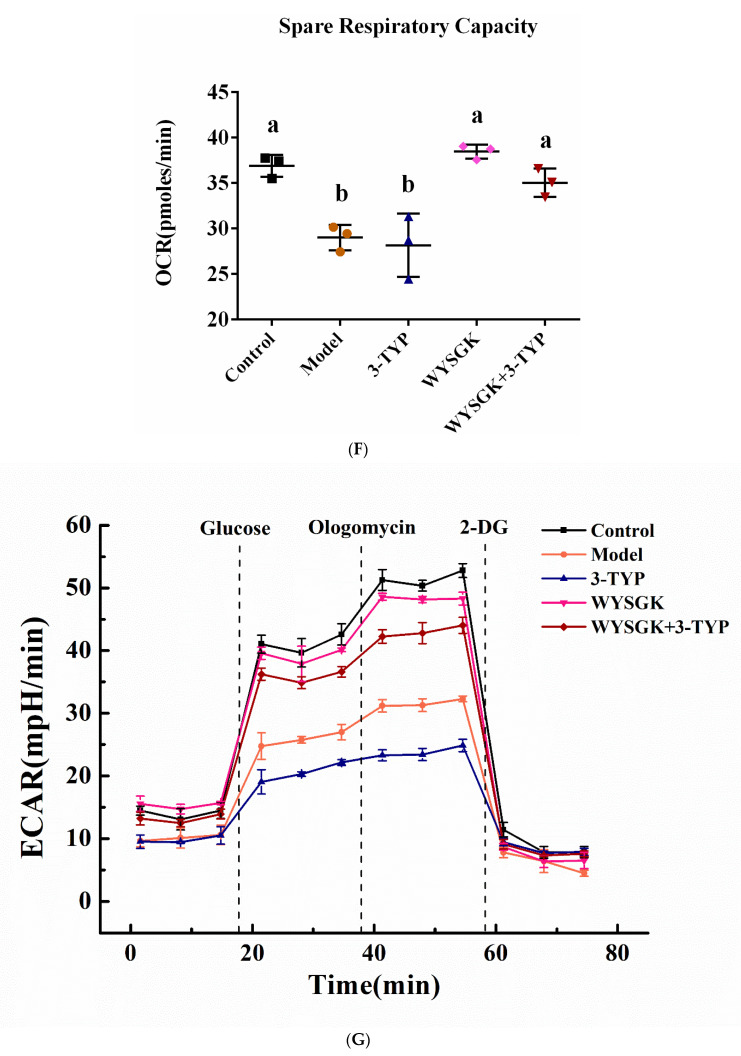

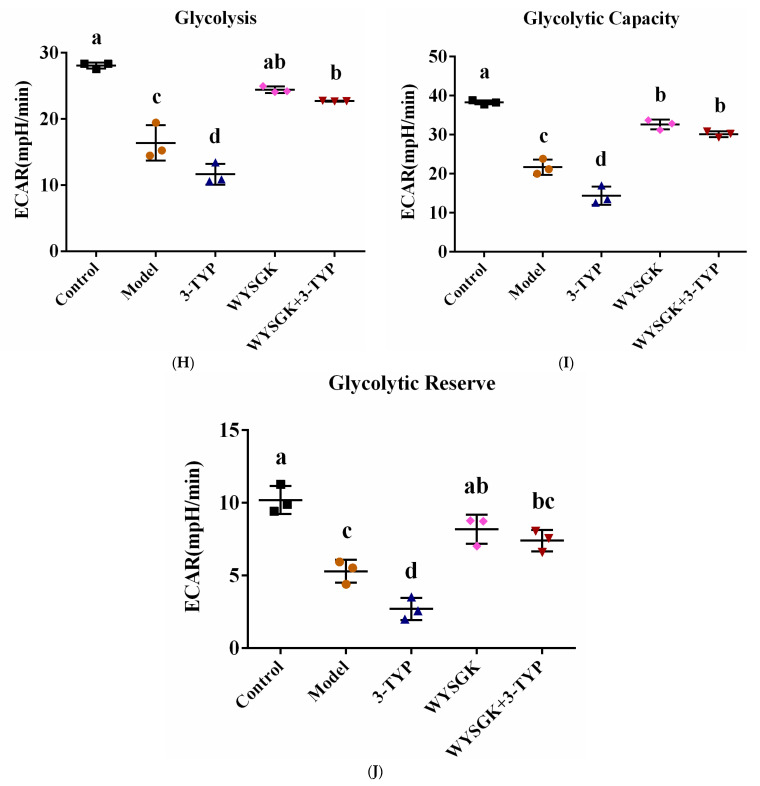
Effects of WYSGK on the cellular oxygen consumption rate (OCR) and extracellular acidification rate (ECAR). Mitochondrial energy metabolism was measured using a Seahorse XF8 extracellular flux analyzer. The mitochondrial (**A**) OCR curve, (**B**) basal respiration, (**C**) ATP production, (**D**) proton leak, (**E**) maximal respiration, and (**F**) spare respiratory capacity. The mitochondrial (**G**) ECAR curve, (**H**) glycolysis, (**I**) glycolysis capacity, and (**J**) glycolysis reserve. Data are presented as means ± SD from three replicates. Different letters denote statistically significant differences (*p* < 0.05).

The Seahorse extracellular flux analyzer was used to evaluate the effect of WYSGK on mitochondrial oxidative phosphorylation and glycolysis in PC12 cells damaged by H_2_O_2_. Figure 5A shows the mitochondrial oxygen consumption rate (OCR) curve. The mitochondrial basal respiratory OCR in PC12 cells damaged by H_2_O_2_ was significantly lower than that in the blank group (*p* < 0.05, Figure 5B). The OCR related to ATP synthesis was detected by oligomycin inhibition of mitochondrial ATP synthase, and the results showed ATP production and proton leakage of the model group cells to be significantly reduced, on an average (*p* < 0.05, Figure 5C,D). Similarly, the maximum respiration tested by uncoupling the electron transfer chain with carbonyl cyanide-p-trifluoromethoxyphenylhydrazone was lower in the model group than in the blank group (Figure 5E). Concurrently, compared to the control group, mitochondria of the model group cells showed a lower reserve capacity to combat energy deficiency (Figure 5F). There was no significant difference in the corresponding indicators between the 3-TYP treatment and the model groups. This could be due to the significant downregulation of SIRT3 levels during H_2_O_2_ damage of PC12 cells, as shown in our previous studies. Compared to that in the model group, basal respiration, ATP production, proton leakage level, maximum respiration, and standby respiration capacity were significantly increased after WYSGK treatment (*p* < 0.05). In the WYSGK+3-TYP treatment group, we observed that when SIRT3 levels were inhibited, the regulation of maximum respiratory capacity and ATP production by WYSGK was also inhibited. Regulation of the mitochondrial oxidative phosphorylation pathway by WYSGK was speculated to be possibly dependent on SIRT3; however, this would need to be confirmed in future studies. Studies have shown that SIRT3 alleviates energy metabolism dysfunction in cells and improves mitochondrial function. We analyzed the extracellular acidification rate of PC12 cells (Figure 5G). Compared to that in the blank group, the basic glycolytic respiration level, glycolytic capacity, and glycolytic reserve value of the model group were significantly reduced (*p* < 0.05, Figure 5H–J), which could be due to imbalance of the redox system after H_2_O_2_-induced cell damage, leading to abnormal mitochondrial energy metabolism. This was consistent with the findings of Lin et al., which revealed that cellular redox imbalance reduces the maximum glycolysis rate and glycolysis capacity of mitochondria [37]. In addition, oxidative stress caused neurotoxicity and irreversible impairment of mitochondrial function, leading to insufficient energy supply. Compared to that in the model group, the basic glycolysis level, capacity, and reserve value of WYSGK increased significantly (*p* < 0.05). This showed that WYSGK has a protective effect on the energy metabolism disorder caused by H_2_O_2_ damage to the cells. Compared to the WYSGK group, the WYSGK+3-TYP treatment group showed no significant difference in glycolysis pathway regulation; WYSGK regulation of the glycolysis pathway was speculated to not depend on SIRT3. According to our current findings, WYSGK can regulate mitochondrial oxidative phosphorylation and glycolysis pathways, and the process of aerobic respiration may depend on SIRT3. Our result was consistent with a previous study, in which SIRT3 was reported to regulate mitochondrial function and biogenesis and protect against mitochondrial damage [38].

## 4. Conclusions

The present study identified that, based on the results of molecular docking, pine nut-derived peptides WYFGK and WYSGK can stably bind to the active region of SIRT3. The SIRT3-WYSGK complex involved π–π interactions and a sandwich structure (Arg-158)/(WYSGK)/(His-248). Antioxidant activity of the derived peptide WYSGK was significantly higher than that of the pine nut peptide WYPGK, which could be associated with the increase in β-sheet content and the active hydrogen atom-produced chemical shifts. The results provided new insights into the effect of amino acids located within an active peptide, which could eventually affect the antioxidant activity of the peptide, possibly by influencing the neighboring amino acids to change the secondary structure of the peptide, the position of active hydrogen atoms, and steric hindrance. We speculated that the smaller side-chain structure of amino acids located inside the peptide chain might facilitate better antioxidant activity. In addition, studies on mitochondrial ultrastructure and respiration suggested that WYSGK can reduce H_2_O_2_-induced mitochondrial damage and alleviate mitochondrial oxidative phosphorylation and glycolysis dysfunction. Notably, the regulation of mitochondrial oxidative phosphorylation by WYSGK was found to depend on SIRT3, which would need to be explored further in future studies. The characterization of pine nut-derived peptides would help clarify the relationship between peptide structure and antioxidant activity and support the application of peptides as functional food ingredients.

## Figures and Tables

**Table 1:** Binding energy of pine nut-derived peptides docking with SIRT3.

Number	Sequence	Binding Energy (kcal/mol)	Hydrogen Bonds Number	π-π Interactions Number	Intermolecular Energy (kcal/mol)	Internal Energy (kcal/mol)	Torsional Energy (kcal/mol)
1	WYDGK	−3.59	8	1	−10.75	−3.66	7.16
2	WYEGK	−1.44	4	1	−8.90	−3.99	7.46
3	WYKGK	−1.94	6	1	−9.69	−4.41	7.76
4	WYRGK	−3.95	8	-	−11.41	−1.76	7.46
5	WYHGK	−3.64	3	-	−10.50	−6.53	6.86
6	WYGGK	−4.34	6	-	−10.60	−3.63	6.26
7	WYAGK	−4.29	6	-	−10.56	−6.42	6.26
8	WYLGK	−4.32	4	-	−11.18	−2.36	6.86
9	WYIGK	−5.37	5	-	−12.23	−4.31	6.86
10	WYVGK	−5.40	2	-	−11.96	−6.08	6.56
11	WYFGK	−6.08	4	-	−12.94	−5.29	6.86
12	WYPGK	−4.66	2	-	−10.63	−6.75	5.97
13	WYMGK	−4.84	5	1	−12.00	−4.12	7.16
14	WYWGK	−5.16	5	-	−12.03	−5.93	6.86
15	WYSGK	−5.87	6	1	−12.73	−6.07	6.86
16	WYQGK	−4.97	4	-	−12.13	−5.75	7.16
17	WYTGK	−4.72	6	-	−11.58	−4.66	6.86
18	WYCGK	−5.18	2	-	−12.04	−4.36	6.86
19	WYNGK	−4.73	8	-	−11.59	−5.11	6.86
20	WYYGK	−3.43	2	-	−10.59	−4.18	7.16

## Data Availability

The data presented in this study are available in article.
